# Safety and efficacy of a device to narrow the coronary sinus for the treatment of refractory angina: A single-centre real-world experience

**DOI:** 10.1007/s12471-016-0862-2

**Published:** 2016-06-14

**Authors:** M. Abawi, F. Nijhoff, P.R. Stella, M. Voskuil, D. Benedetto, P.A. Doevendans, P. Agostoni

**Affiliations:** 1 Department of Cardiology, University Medical Center Utrecht, Utrecht, The Netherlands; 2 University of Milan, Milan, Italy; 3 Department of Cardiology, St. Antonius Hospital Nieuwegein, Nieuwegein, The Netherlands

**Keywords:** Stable angina pectoris, Refractory angina, Coronary sinus, Reducer stent

## Abstract

**Objective:**

The coronary sinus Reducer is a recently introduced device to treat patients with severe angina symptoms refractory to optimal medical therapy and not amenable for conventional revascularisation. We aimed to assess the safety and efficacy of the Reducer in a real-world cohort of patients with refractory angina.

**Methods:**

This is a single-centre retrospective registry. Patients with severe angina symptoms, objective evidence of myocardial ischaemia using any adequate non-invasive modality and without options for conventional revascularisation were regarded eligible for Reducer implantation.

**Results:**

Twenty-three patients (74 % male, mean age 70 ± 8 years, 91.3 % previous bypass surgery, 82.6 % previous percutaneous intervention, 47.8 % previous myocardial infarction, 52.2 % diabetes mellitus) underwent Reducer implantation. The safety endpoint (successful implantation of the first device without device-related adverse events) was met in all patients. After a median follow-up of 9 (8–14) months the efficacy (any reduction in Canadian Cardiovascular Society (CCS) class and revascularisation-free survival) was reached in 17 patients (74 %): 8 patients (34.8 %) improved by 1 CCS class, 7 (30.4 %) by 2 CCS classes and 2 (8.7 %) by 3 CCS classes. One patient died 4 months after implantation because of progressive heart failure (not associated with Reducer implantation).

**Conclusion:**

In this single-centre real-world experience, Reducer implantation was safe and demonstrated excellent clinical efficacy in the treatment of refractory angina at mid-term follow-up.

## Introduction

Despite the large armamentarium of anti-ischaemic medical therapies and revascularisation techniques, a considerable number of patients suffer from angina pectoris which is refractory to medical treatment and related to coronary artery disease (CAD) ineligible for conventional revascularisation [1, 2]. In the light of the ageing population and the increased life expectancy of CAD patients, the global prevalence of refractory angina is expected to rise [2–4]. According to available data, refractory angina is diagnosed in 5 to 15 % of coronary angiography candidates, which implies approximately 30–50,000 new patients each year in Europe [2], more than 500,000 in Canada and 1.8 million in the United States [5], although robust epidemiological data are lacking. Patients with refractory angina are frequently hospitalised, use many anti-ischaemic drugs, experience limited quality of life because of debilitating symptoms, and are often strikingly young (with a mean age 65–70) [1, 6]. Therefore, treatments that relieve angina symptoms and improve quality of life are urgently needed for these no-option patients. A broad array of therapies (e. g. novel medical treatment such as ivabradine and ranolazine, internal mammary artery implants, extracorporeal shockwave therapy, spinal cord stimulation, transmyocardial laser revascularisation, gene therapy, cell therapy) have been investigated, none of which have become mainstream [6–9].

Recently, the coronary sinus (CS) Reducer was introduced into clinical practice as a new device-based treatment for patients with refractory angina. This balloon-expandable stainless steel stent is designed to increase coronary venous pressure by creating a focal stenosis in the CS following its implantation. Coronary venous pressure elevation alleviates angina by improving perfusion of the ischaemic regions of the myocardium by increasing coronary collateral blood flow [10, 11], redirecting flow to the endocardium [11, 12], and possibly stimulating myocardial neovascularisation [13]. The principle of enhancing myocardial perfusion by elevating coronary venous pressure was already described in 1936 by Gross and colleagues [14] and was translated into clinical practice in 1954 by Beck and Leighninger [15], who performed surgical partial ligation of the CS in patients with angina pectoris (as part of the Beck I operation).

Reducer implantation is the percutaneous equivalent of surgical narrowing of the CS and shows promise as treatment for refractory angina. Two small prospective registries demonstrated a symptomatic improvement in the majority (~85 %) of Reducer-treated patients, accompanied by a reduction in objective measures of myocardial ischaemia in a subset of patients [13, 16]. Follow-up data indicated durability of these results for up to 3 years [17]. The recently published COronary SInus Reducer for treatment of refractory Angina (COSIRA) trial firmly substantiated the evidence base of CS narrowing by Reducer implantation as treatment for refractory angina [18]. In this prospective, multicentre, double-blind, sham-controlled trial the reduction of symptoms, in terms of the percentage of patients whose Canadian Cardiovascular Society (CCS) functional class improved as well as their quality of life, was significantly larger in the treatment group compared with the sham-controlled group after a 6-month follow-up period [18].

Since 2014, Reducer implantations are performed in our institution as part of clinical care. The aim of the present study is to evaluate the safety and efficacy of CS Reducer implantation in a real-world cohort of patients with refractory angina.

## Methods

This is a single-centre, single-arm, retrospective study. Patients were regarded as eligible for Reducer implantation when meeting the following criteria: 1) symptomatic angina despite optimal pharmacological therapy; 2) objective evidence of inducible myocardial ischaemia, as determined by bicycle stress electrocardiography, dobutamine stress echocardiography, single-photon emission computed tomography (SPECT), or stress magnetic resonance imaging; and 3) proven CAD of the left coronary artery not amenable for percutaneous coronary intervention (PCI) or coronary artery bypass grafting (CABG), because of unsuitable coronary anatomy, diffuse atherosclerosis, or the absence of satisfactory landing zones for bypass grafting, according to the decision of the local heart team.

When compared with the strict study enrolment criteria of the COSIRA trial [18], no formal exclusion criteria were considered for this real-world registry besides the presence of ischaemia related exclusively to the right coronary artery (also an exclusion criterion in COSIRA). As opposed to the COSIRA trial, in which only patients with a positive dobutamine stress echocardiography were included, any form of proven ischaemia was accepted. Moreover, the presence of pacemaker leads in the right heart was allowed. Only patients with hardware already present in the CS (a left ventricular pacemaker wire for cardiac resynchronisation therapy (CRT)) were excluded. In our institution 4 patients were included in the COSIRA trial: 2 in the treatment arm and 2 in the sham arm. The 2 COSIRA participants from the sham arm were included in the current registry as they underwent Reducer implantation after the primary endpoint of the COSIRA study was reached (6-month follow-up). The 2 COSIRA participants receiving the Reducer in the trial were not included in the current registry.

Angina status was evaluated by an independent physician, who contacted all patients at follow-up by telephone. Other relevant clinical endpoints, including death by any cause, myocardial infarction and revascularisation, were also recorded. The safety endpoint was defined as the successful delivery of the first device in the proper location and without any device-related adverse events. The efficacy endpoint was defined as any reduction in CCS class and revascularisation-free survival. All patients gave informed consent for the procedure, and because of the retrospective nature of the study, the requirement of approval from the ethics committee was waived.

## Device and implantation procedure

The Reducer is a percutaneous, endo-luminal, hourglass-shaped, balloon-expandable stainless steel stent that is designed for implantation in the CS to create a focal stenosis. The device received CE approval for the treatment of refractory angina in November 2011. It is available in one single size that is suitable for a broad range of CS anatomies (with a diameter between 9 and 13 mm).

**Fig. 1:**
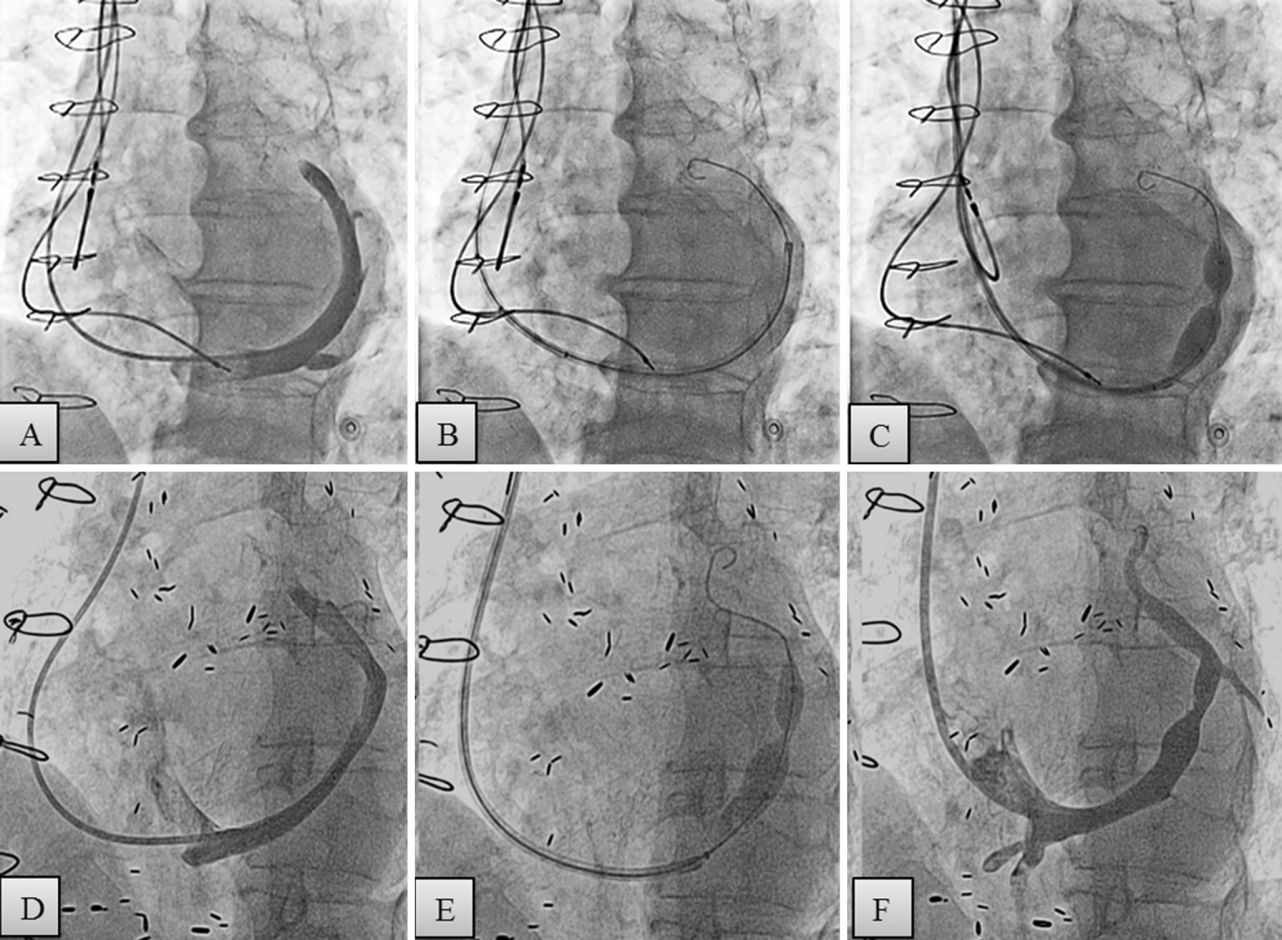
Reducer implantation in patients with (**A–C**) and without presence of Dual-Chamber leads (**D–F**). Angiogram of the coronary sinus (**A** and **D**), 9 French guiding catheter positioned deeply in the coronary sinus (**B**) implantation of the Reducer after retrieval of the 9 French guiding catheter to the proximal part of the coronary sinus (**C** and **E**), angiographic control of the coronary sinus after Reducer implantation (**F**)

All procedures were performed by one interventional cardiologist (PA) with experience in Reducer implantation. The right jugular vein was punctured under local anaesthesia and 6 French multipurpose or Amplatz left diagnostic catheter was introduced to selectively cannulate and image the CS under fluoroscopic guidance. Once the CS was deemed suitable for Reducer implantation, a 9 French guiding catheter was advanced in the distal part over a 0.035 inch guide wire with support of the diagnostic catheter (mother-and-child technique). The Reducer, which comes preloaded on the balloon, was then introduced over the wire into the CS and positioned at the desired site. By gentle retrieval of the guiding catheter, the Reducer was exposed and implanted by inflating the delivery balloon. The inflation pressure ranged between 2 and 6 atm according to the diameter of the CS. An additional contrast injection via the guiding catheter during inflation of the Reducer helped to confirm slight oversizing as compared with the diameter of the CS (Fig. 1).

The procedure was performed in a day-hospital setting. Patients were discharged once proper closure of the access site was confirmed. All patients continued their baseline anti-anginal medication. Double antiplatelet therapy with aspirin and clopidogrel was recommended for 1 month. In case of oral anticoagulant therapy, additional clopidogrel was mandated for 1 month.

## Statistical analysis

Categorical variables are expressed as frequency and percentages. Continuous variables are expressed as mean and standard deviation if normally distributed or as median (interquartile range). Baseline and clinical follow-up data were compared using the Wilcoxon signed-rank test. A two-tailed probability value of < 0.05 was considered statistically significant. All statistical analyses were carried out using the IBM Statistical Package for Social Science for Windows, version 21.0 (IBM Corp., Armonk, NY, USA) and GraphPad Prism, version 6.

## Results

In 2014, 23 consecutive patients (74 % male, mean age 70 ± 8 years) with medically refractory angina without conventional revascularisation options were electively treated with the Reducer. One additional patient was refused further screening for possible Reducer implantation because of cardiac resynchronisation therapy hardware already present in the CS. The majority of patients had a history of revascularisation by means of PCI (82.6 %) and/or CABG (91.3 %). The incidence of diabetes mellitus (52.2 %) and prior myocardial infarction (47.8 %) was high. Despite being on optimal anti-anginal treatment patients were severely symptomatic, suffering from CCS class 3 to 4 symptoms, except for one patient who had CCS 2 angina symptoms. According to nuclear imaging data (available for the majority of the patients), the quantification of ischaemia was as follows: mean summed stress score of 11.94 ± 7.18, summed rest score of 6.69 ± 7.54 and summed differential score of 6.41 ± 4.61. The safety endpoint (successful delivery and deployment at the planned implant site without any device-related complications) was met in all patients. There were no cases of device migration or cases of pericardial tamponade. One patient suffered an access site haematoma, which was successfully treated in a conservative way. The patient was hospitalised for observation for one night. Baseline and procedural characteristics of the study population are summarised in Tab. 1 and 2. Data for each individual case are presented in Tab. 3.

**Tab. 1:** Baseline characteristics of patients receiving coronary sinus Reducer

	Overall (*n* = 23) (*n* (%))
Age, years	70 ± 8
Gender, male	17 (74)
Body mass index, kg/m^2^	29 ± 4
Body surface area, m^2^	2.0 ± 0.2
Diabetes mellitus	12 (52.2)
Hypercholesterolaemia	13 (56.5)
Hypertension	14 (61.1)
Current or previous smoking	16 (69.5)
Familial coronary artery disease	20 (86.9)
Prior myocardial infarction	11 (47.8)
Prior percutaneous coronary intervention	19 (82.6)
Prior coronary artery bypass graft	21 (91.3)
Presence of pacemaker	4 (17.4)
Left ventricle ejection fraction, %	59 ± 9
Glomerular filtration rate, ml/min	68 ± 19
*Canadian Cardiovascular Society angina class*	
2	1 (4.3)
3	12 (52.2)
4	10 (43.4)
*Baseline medication use*	
Long-acting nitrates	18 (78.2)
Calcium-channel antagonists	12 (52.2)
Beta-blockers	20 (86.9)

**Tab. 2:** Periprocedural outcomes

	Overall (*n* = 23)
*Preprocedural data*	
*Coronary artery disease (untreated)*	
Diffuse microvascular problem	2 (8.6)
1-vessel disease	12 (52.2)
2-vessel disease	7 (30.4)
3-vessel disease	2 (8.6)
*Location of myocardial ischaemia (multiple territories allowed)*	
Anterior	15 (65.2)
Inferior	8 (34.7)
Lateral	15 (65.2)
Apical	4 (17.4)
Septal	8 (34.7)
*Procedural outcomes*	
Procedural time, min	27 ± 4
Contrast volume, ml	52 ± 19
Radiation, mGy	480 ± 331
Fluoroscopy time, min	10 [8–17]
Access site complication, *n* (%)	1 (4.3)
Device embolisation, *n* (%)	0
Cardiac tamponade, *n* (%)	0
Intra-procedural death, *n* (%)	0
Safety endpoint^a^, %	100
*Post-procedural outcomes*	
Creatine kinase-MB, µg/l	3.8 ± 1.9
High sensitivity troponin, ng/l	298 ± 290

^a^ successful placement of the device in the coronary sinus without any periprocedural adverse events

**Tab. 3:** Individual patients characteristics

**Patients characteristics at baseline**								**Clinical outcomes at follow-up**	
*N*	*Gender*	*Age*	*Ivabradine use*	*Presence of pacemaker*	*Ejection fraction (%)*	*Location of ischaemia*	*CCS*	*Clinical events*	*CCS*
1^a^	Female	68	–		71	Anteroseptal-apical^b^	3	–	1
2	Female	69	–		63	Anterolateral^b^	4	–	3
3^a^	Male	48	–		54	Inferolateral^b^	4	Repeat revascularisation	2
4	Male	76	–		59	Inferolateral^b^	2	Repeat revascularisation	2
5	Male	82	–		65	Inferior ^c^	3	–	2
6	Male	65	–		62	Anterolateral^b^	3	–	1
7	Male	62	–	Dual-Chamber	40	Anterolateral^b^	3	–	2
8	Male	66	Yes		59	Anteroapical^b^	4	–	3
9	Female	76	–	Dual-Chamber	67	Anteroseptal-apical^b^	4	–	3
10	Male	75	–		64	Anteroseptal-apical- inferior^b^	3	–	1
11	Male	51	–		60	Anteroinferolateral^b^	4	–	1
12	Female	78	–		65	Anterolateral^b^	3	–	2
13	Female	62	Yes		65	Anteroseptal-lateral^b^	3	–	1
14	Male	73	–		50	Anterolateral^b^	3	Death	3
15	Male	75	–		46	Inferolateral-anteroseptal^b^	4	–	3
16	Male	72	–		51	Inferolateral^b^	3	–	1
17	Female	80	–		69	Lateral^b^	3	–	1
18	Male	80	–	Dual-Chamber	52	Anterolateral^b^	4	–	4
19	Male	66	Yes		70	Anteroseptal^b^	3	–	1
20	Male	71	–		61	Anteroseptal^b^	3	–	3
21	Male	69	–		45	Septal^b^	4	–	1
22	Male	68	–	Dual-Chamber	50	Inferolateral^d^	4	–	3
23	Male	73	–		60	Lateral^b^	4	–	4

^a^ patients from current study who also participated in the control-arm of the COSIRA trial. Ischaemia detection method: ^b^ SPECT; ^c^ stress echocardiography; ^d^ stress magnetic resonance. *CCS* Canadian Cardiovascular Society functional class

**Fig. 2:**
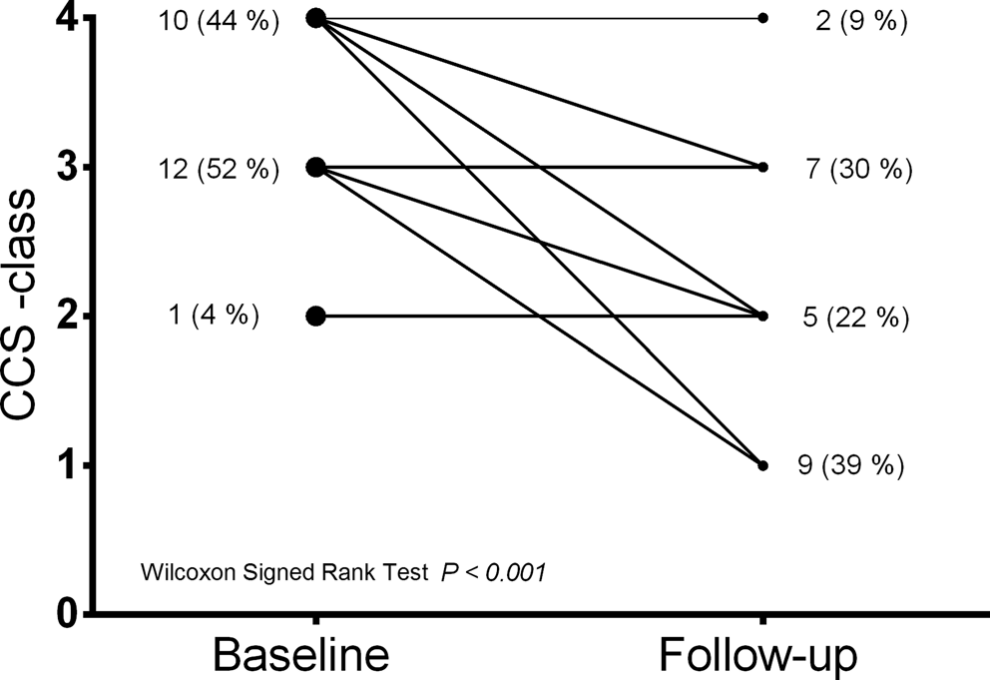
Canadian Cardiovascular Society (CCS) class of individual patients at baseline and 9 months after Reducer implantation

**Fig. 3:**
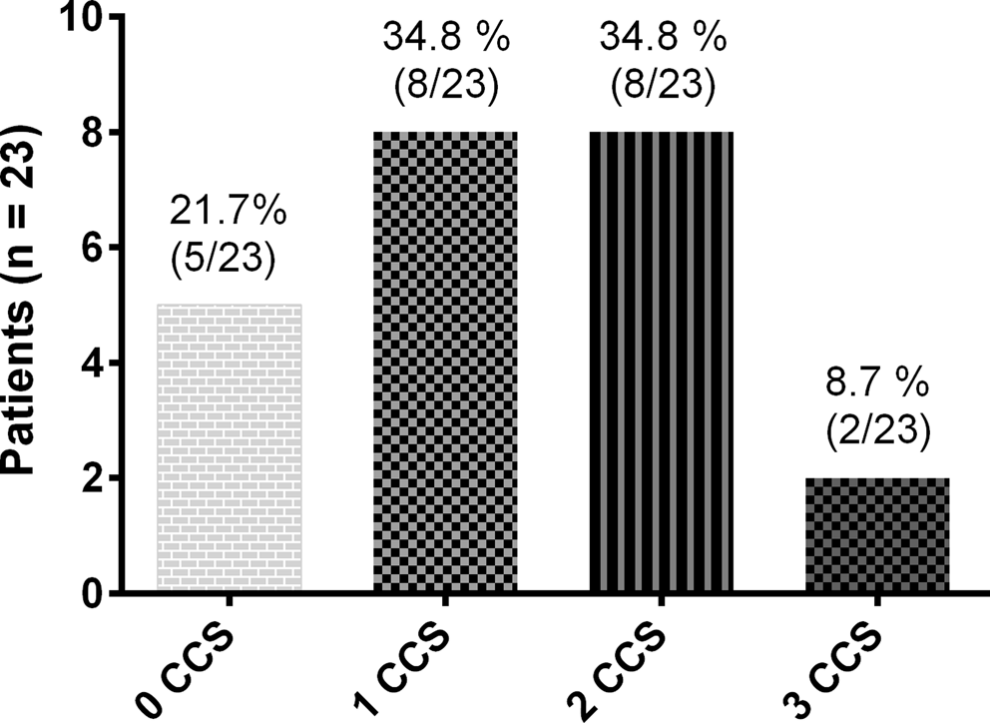
Canadian Cardiovascular Society (CCS) functional class changes after Reducer implantation (positive numbers mean improvement). According to this figure, 17 patients improved at least 1 CCS class

After a median follow-up of 9 (8–14) months, the mean CCS class was reduced from 3.4 ± 0.6 at baseline to 2.1 ± 1.0 at follow-up (*p* < 0.001). The efficacy (any reduction in CCS class with revascularisation-free survival) was reached in 17 patients (74 %): 8 patients (34.8 %) had improved by 1 CCS class, 7 (30.4 %) by 2 CCS classes and 2 (8.7 %) by 3 CCS classes. Failure to comply with the efficacy was due to the absence of symptomatic improvement in 5 patients (21.7 %) and repeat revascularisation in 1 patient (4.3 %) (Fig. 2 and 3, Tab. 3). Of the 5 patients with no change in CCS class, one underwent PCI of a saphenous vein graft to the right coronary artery at the level of the anastomosis with the right descending posterior artery 6 months after Reducer implantation. This lesion was not visible on the coronary angiogram performed 1 year before Reducer implantation. One patient (with temporary improvement in symptoms, but recurrence of angina) underwent revascularisation of a recurrent in-stent chronic total occlusion of an aberrant left main coronary artery 9 months after Reducer implantation. This lesion was already known before Reducer implantation, but was deemed extremely complex to treat. Left main PCI was uneventful and the patient improved by 2 CCS classes. Another patient whose symptoms did not improve after Reducer implantation died due to progressive heart failure at 4‑month follow-up. No autopsy was performed.

## Discussion

In the present study we evaluated the safety and efficacy of Reducer implantation in a real-world cohort of patients with angina symptoms refractory to optimal medical therapy in the presence of CAD not amenable for revascularisation. All devices were implanted in the proper position without any device-related adverse events. In particular, no periprocedural complications occurred in patients with pacemaker leads (*n* = 4). Efficacy was high as the majority (74 %) of patients demonstrated symptomatic improvement free from additional revascularisation at a median follow-up of 9 months.

The efficacy observed in this real-world population is in line with prior studies. Any improvement in CCS class was observed in 74 % of patients, which is intermediate of the ~85 % reported in previous non-randomised studies [13, 16] and the 71 % in the treatment arm of the COSIRA trial [18]. The magnitude of angina alleviation was also fairly comparable. Symptom reduction of ≥ 2 CCS classes occurred in 39 % and 35 % of patients in the present study and COSIRA, respectively, but was slightly more frequent in the previous non-randomised studies, as 53 % and 45 % of patients improved ≥ 2 CCS classes [13, 16]. Similar effectiveness of the Reducer across the studies was reflected in the mean CCS class at baseline and follow-up: 3.4 ± 0.6 vs. 2.1 ± 1.0 in this study, 3.2 ± 0.4 vs. 2.1 ± 1.0 in COSIRA, and 3.1 vs. 1.6 and 3.3 ± 0.6 vs. 2.0 ± 1.0 in the aforementioned registries [13, 16].

The observed treatment effect of Reducer implantation may be partially attributable to a placebo effect, especially when considering the subjective nature of the efficacy endpoint. In double-blinded trials evaluating refractory angina therapies, up to 41 % of patients receiving placebo have been reported to improve ≥ 2 CCS classes [6]. In order to reliably contrast Reducer therapy with the placebo effect of a sham procedure, extensive precautions were taken in the pivotal COSIRA trial to maintain double-blind conditions [19]. Impressively, the COSIRA trial still succeeded in showing a clear benefit of Reducer implantation over the sham procedure. Significantly more patients in the Reducer group had symptomatic improvement of ≥ 2 CCS classes (35 % vs. 15 %, *p* = 0.02), as well as improvement of ≥ 1 CCS class (71 % vs. 42 %, *p* = 0.003) at 6 months. Moreover, the gain in quality of life as evaluated by the Seattle Angina Questionnaire was more pronounced (17.6 vs. 7.6 points, *p* = 0.03) [18]. The unequivocal results of COSIRA strongly suggest effectiveness of Reducer implantation, but confirmation in larger trials is required as the sample size was relatively small (*n* = 104).

The mechanisms underlying the reduction of angina symptoms after Reducer implantation are not fully understood. However, two potential anti-ischaemic effects of elevation of CS pressure have been hypothesised, both related to the increased back pressure in the venules and capillaries caused by CS pressure elevation. Several studies observed increased flow in pre-existing collaterals between the non-ischaemic myocardium and ischaemic myocardium after CS narrowing/occlusion [10, 11, 20]. Enhanced coronary collateral flow may result from the back pressure exerted resistance to coronary flow into the non-ischaemic myocardium, leading to a shift of flow to the low pressure surroundings of the ischaemic myocardium [18]. Other studies also suggest that elevated back pressure promotes blood flow to the ischaemia-prone endocardium [11, 12]. Endocardial blood flow is often diminished in the presence of epicardial coronary stenosis because of decreased perfusion pressure and dysfunctional physiological mechanisms to preserve endocardial flow during exercise (i. e. the lack of sympathetically mediated constriction of subepicardial vessels) [21]. Increased back pressure lowers resistance in the endocardial vascular bed by inducing dilatation of subendocardial capillaries, which increases flow and restores the endocardial-to-subepicardial flow ratio. Both increased collateral flow and redistribution of flow to the endocardial layers of the myocardium reduce ischaemia and may be responsible for angina alleviation after Reducer implantation. On the long run, neovascularisation may also be involved in the anti-ischaemic effect of Reducer therapy. This was suggested in a classic histological study that reported an ‘unusual degree of vascularity’ in ischaemic myocardium after the Beck II operation (arterialisation of the CS followed by partial ligation) in a canine coronary occlusion model [22].

The notion that alleviation of angina by Reducer implantation involves a true reduction in myocardial ischaemia is reinforced by the significant changes in objective measures of ischaemia that have been observed [13, 16]. Albeit in small numbers of patients, a significant decrease in ST-segment depression during exercise tests, wall motion abnormality scores on stress echocardiography, and defect extent scores on SPECT have been detected 6 months after the procedure [13, 16]. In COSIRA there was only a trend towards improved ischaemic parameters in the treatment group, as the study was not powered to detect differences in this endpoint [18]. The anti-ischaemic potential of elevation of CS pressure is also apparent in other CS interventions. Intermittent CS occlusion has been shown to provide myocardial salvage during coronary occlusion, reducing myocardial ischaemia severity [20] and infarct size [23]. However, the beneficial effects of intermittent CS occlusion involve other mechanisms besides CS pressure elevation, including reopening of the microvasculature, wash out of toxic metabolites, and activation of regenerative processes [24].

The absence of symptomatic improvement, observed in 15 to 30 % of cases [13, 16, 18], may be explained by unfavourable coronary venous anatomy. Ubiquitous presence of Thebesian veins, draining directly into the ventricular chambers, may prevent coronary venous pressure from rising despite adequate narrowing of the CS [25].

Narrowing of the CS by Reducer implantation seems a safe procedure as no device-related adverse events were documented in our study. The only non-device related complication comprised a relatively harmless access-site haematoma. No device displacements occurred during the procedure. Although no imaging studies were performed to confirm preserved device position and stent patency at follow-up, late migration or occlusion seems unlikely [26], as demonstrated before by serial computed tomography evaluation [13]. The absence of safety issues specifically related to Reducer therapy is in accordance with previous studies [13, 16, 18].

Patients treated in the current study had an average left ventricle fraction ejection of 59 ± 9 % without patients with ejection fraction < 35 %, thus CRT implantation is definitely not indicated in any of them. If necessary, it is easy to dilate the narrowed central segment of the stainless steel Reducer device with a simple balloon-dilatation technique. This approach may allow future passage of a CS lead for CRT.

## Limitations

This study has some important limitations. In the first place, its retrospective single-centre design and small sample size. Furthermore, the largely subjective nature of the efficacy (involving change in CCS class) and the lack of ischaemia detection at follow-up to support the symptomatic improvement with reductions in objective measures of ischaemia. Finally, we did not provide a standard medical treatment control group.

## Conclusion

In this single-centre real-world experience, Reducer implantation was safe and efficacious, as the majority of refractory angina patients demonstrated symptomatic improvement at mid-term follow-up. Larger trials are eagerly awaited to further build the evidence base of Reducer implantation for refractory angina.
